# Supplementary material for: New Trends and Advances in Non-Variceal Gastrointestinal Bleeding—Series II
Source: J Clin Med. 2021 Jul 8;10(14):3045. doi: 10.3390/jcm10143045 (PMC8303152; doi:10.3390/jcm10143045)

Supplementary Figure S2: Diagram to summarize the management of the lower gastrointestinal bleeding.

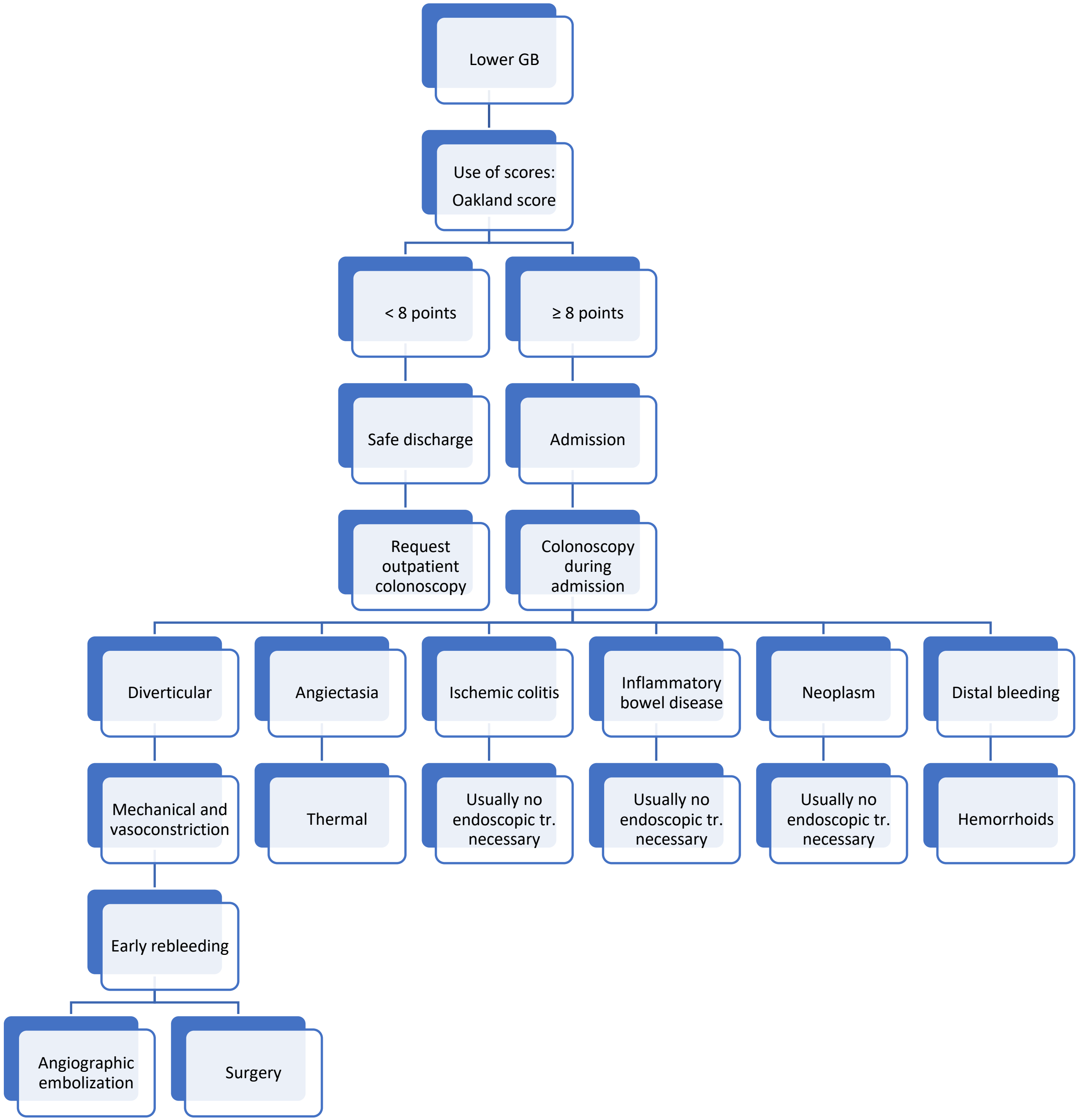

Supplement: Supplementary file 1 [file jcm-10-03045-s001.zip › jcm-1275936 Figure S2-Management LGB.pdf]
